# Humoral response against host-mimetic homologous epitopes of *Mycobacterium avium* subsp. *paratuberculosis* in Japanese multiple sclerosis patients

**DOI:** 10.1038/srep29227

**Published:** 2016-06-30

**Authors:** Davide Cossu, Kazumasa Yokoyama, Leonardo Antonio Sechi, Shigeru Otsubo, Yuji Tomizawa, Eiichi Momotani, Nobutaka Hattori

**Affiliations:** 1 Juntendo University School of Medicine, Department of Neurology, Tokyo, 113-8421, Japan; 2 Sassari University, Department of Biomedical Sciences, Sassari, 07100, Italy; 3 Sangenjaya hospital, Department of Blood Purification, Tokyo, 154-0024, Japan; 4 Tohto College of Health Sciences, Department of Human-care, Saitama, 366-0052, Japan

## Abstract

Several works have demonstrated the existence of a link between *Mycobacterium avium* subsp. *paratuberculosis* (MAP) and MS in Italy. In this study, we analyzed the serology of MAP in a Japanese population while looking at several markers of MAP. Fifty MS patients, 12 clinically isolated syndrome (CIS) patients, 30 other neurological disorders (OND) patients, and 50 healthy controls (HCs) were tested using ELISA for the presence of IgG antibodies toward immunodominant epitopes MAP_0106c_121-132_, homologues MBP_85-98_, homologues IRF5_424-432_, MAP_4027_18-32,_ and MAP_2694_295-303_. MAP-positive patients were also analyzed in relation to their clinical/demographic characteristics. Amongst all peptides, only antibodies against MAP_2694_295-303_ were more prevalent in MS patients (30%), as compared to OND patients (3%) (p = 0.009; area under roc curve (AUC) = 0.61) and HCs (2%) (p = 0.0004; AUC = 0.65) and in CIS patients (25%) compared to HCs (p = 0.023; AUC = 0.55). Logistic regression analysis showed a higher frequency of anti-MAP_2694_295-303_ antibodies in the sera of oligoclonal bands positive MS patients (p = 0.2; OR = 2, 95%CI: 0.55–7.7). These findings support the view that MAP could act as a risk factor or a triggering agent of MS in some Japanese patients with a genetic susceptibility to the mycobacterium.

Multiple sclerosis (MS) is a chronic inflammatory demyelinating disorder affecting the central nervous system (CNS). The etiology of MS is still unknown but both genetic and environmental factors appear to play important roles in conferring susceptibility to the disease^1^. A common hypothesis states that during a systemic bacterial or viral infection, the innate and adaptive immune systems in the CNS involve many molecules that could induce a variety of neurological disorders such as MS^2^. Among the existing potential infectious risk factors related to MS, *Epstein-Barr* virus (EBV) is the most important candidate trigger^3^. Another interesting candidate is *Mycobacterium avium* subsp. *paratuberculosis* (MAP), an intracellular bacterium responsible of Johne’s disease in ruminants that has also been associated with several autoimmune diseases such as MS^4^. The exact role of MAP in MS pathogenesis is unclear, but current studies suggests that both MAP and EBV infection might be able to elicit MS related autoimmunity, most likely acting through a common target^5^,^6^,^7^. In particular, antigens deriving from MAP could be cross-recognized by antibodies (Abs) targeting self-epitopes through a molecular mimicry mechanism^6^,^7^. The association between MAP and MS was established for the first time in Sardinia^8^, an Italian Mediterranean island that shows one of the highest rate of MS in the world (total crude prevalence was 260.3 per 100,000 inhabitants in 2009)^9^. Furthermore, more than 60% of Sardinian flocks are infected by MAP^4^. Hence, it has been hypothesized that the wide presence of potentially infective microorganisms such as MAP and EBV could act synergistically (through common targets) in developing MS in genetically predisposed individuals^8^.

Comparison between Caucasian and Asian populations shows that MS is rare in Asian^10^. Regarding the epidemiology of MAP in Japan, the overall farm exposure to mycobacterium is only 2%^11^, but a recent study conducted on 130 healthy people, showed a presence of Abs against MAP surface antigens in the sera of 14% of the subjects involved in the study^11^. Therefore the possibility exist that Japanese people are likely mainly exposed to mycobacterium, through the consumption of MAP-contaminated Westernized dairy products^11^.

Given that recent studies have revealed a general increase of MS incidence in Japan together with a decrease in the registered age at onset^12^, and considering that a clear association with a particular pathogen has not been found, we aimed to investigate whether MAP could be one of the potential infectious agents involved in the triggering of MS in genetically predisposed Japanese individuals by analyzing the humoral response against selected antigenic peptides of the mycobacterium already identified and related to MS^6^,^7^,^13^.

## Results

### Humoral response against MAP-derived and human homologues peptides

We investigated whether MAP and human homologues peptides, which were highly immunogenic in MS subjects from Italy, could be recognized in MS Japanese patients. Five peptides were examined using indirect ELISA in 50 MS patients, 13 clinically isolated syndrome (CIS) patients, 30 other neurological disorders (OND) patients, and 50 healthy controls (HCs).

**Figure 1:**
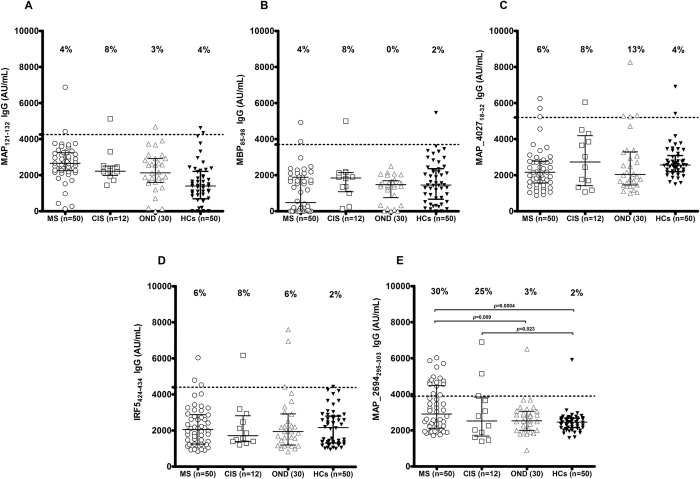
ELISA-based analysis. Fifty patients with multiple sclerosis (MS), 12 patients with clinically isolated syndrome (CIS), 30 patients with other neurological diseases (OND) and 50 healthy controls (HCs) were tested for their reactivity against plate-coated with MAP_0106c_121-132_ (**A**), MBP_85-98_ (**B**) MAP_4027_18-32_ (**C**), IRF5_424-434_ (**D**) and MAP_2694_295-303_ (**E**) peptides. The bars represent the median ± interquartile range, while the percent fraction of antibody positive sera is indicated on top of distribution. Cut-off values for positivity, calculated by ROC analysis, are indicated by dashed lines. P values, significant if <0.05, are indicate by two headed arrows (**E**).

Only MAP_2694_295-303_ peptide was highly recognized, proving detectable reactivity (Fig. 1E). Antibodies against MAP_2694_295-303_ peptide were found in 15 out of 50 MS patients (30%; 95% confidence interval [CI]: 17–43%), in 1 out of 30 OND patients (3%; 95% CI: −3–9%) (Fisher exact test: p = 0.009; AUC = 0.65) and in 1 out of 50 HCs (2%; 95% CI: −2–5%) (p = 0.0004; AUC = 0.70).

Amongst the 15 MS Ab-positive patients, 12 were relapsing remitting, 2 were secondary progressive, and 1 was primary progressive.

High levels of IgG_1_ were observed in 14 out of 15 (93%, 95% CI: 80–106%) sera. IgG_1,_ opsonic Abs that bind with high-affinity FcγRI receptor, are responsible for the antigen uptake by macrophages^14^. One secondary progressive MS patient (7%, 95% CI: −6–20%) was positive for specific IgG_4_, an isotype present after chronic exposure to antigen^11^. A follow-up study demonstrated that in MS patients treatment with glatiramer acetate induced a switch from IgG_1_ to IgG_4_ after 9 months of therapy^15^, hence the possibility exists that a prolonged exposure to MAP antigens may cause a comparable isotype switching from anti-MAP_2694_295-303_ IgG_1_ to IgG_4_, due to the interaction between B cells and T lymphocyte^16^.

No significant levels of IgG_2_ and IgG_3_ were detected in MS sera.

Similar results were obtained in the CIS group, indeed total IgG (IgG_1_ subclass) Abs against MAP_2694_295-303_ were present in 25% (95% CI: 0.5–49%) of CIS patients. Compared with the HCs groups the association is statistically significant (p = 0.023, AUC = 0.65).

No statistically significant results were observed in the 4 other peptides. The homologues MAP_0106c_121-132_ (Fig. 1A) peptides were recognized in 4% (95% CI: −1.4–9.4%) of the MS patients, 8% (95% CI: −7.4–23.2%) of the CIS patients, 3% (95% CI: −3.1–9.1%) of OND patients, and 4% (95% CI: −1.4–9.4%) of the HCs. The MBP_85-98_ (Fig. 1B) peptides were recognized by 4% (95% CI: −1.4–9.4%) of the MS patients, 8% (95% CI: −7.4–23.2%) of the CIS patients, 0% of OND patients, and 2% (95% CI: −2–6%) of HCs.

MAP_4027_18-32_ (Fig. 1C), Abs were detected in 6% (95% CI: −0.6–12.6%) of MS patients, 8% (95% CI: −7.3–23.3%) of CIS patients, 13% (95% CI: −7.3–23.35%) of OND patients, and 4% (95% CI: −1.4–9.4%) of HCs. Homologues IRF5_424-434_ (Fig. 1D) Abs were detected in 6% (95% CI: −0.6–12.6%) of MS patients, 8% (95% CI: −7.3–23.3%) of CIS patients, 6% (95% CI: −2.5–14.5%) of OND patients, and 4% (95% CI: −1.4–9.4%) and 2% (95% CI: −2–6%) of HCs.

The frequencies of Abs recognizing MAP epitopes and human homologues regions, a correlation analysis performed on MS patients showed: a good degree of correlation between MAP_0106c_121-132_ and homologues MBP_85-98_ (r_s_ = 0.43, p = 0.001), a good degree of correlation between MAP_4027_18-32_ and homologues IRF5_424-434_ (r_s_ = 0.42, p = 0.002), a moderate degree of correlation between MAP_0106c_121-132_ and MAP_2694_295-303_ (r_s_ = 0.38, p = 0.005), a moderate degree of correlation between MAP_0106c_121-132_ and MAP_4027_18-32_ (r_s_ = 0.31, p = 0.02), and no correlation between MAP_0106c_121-132_ and MAP_4027_18-32_ (r_s_ = 0.43, p = 0.001).

### Comparison between Ab-titer between Italian and Japanese MS subjects

**Figure 2:**
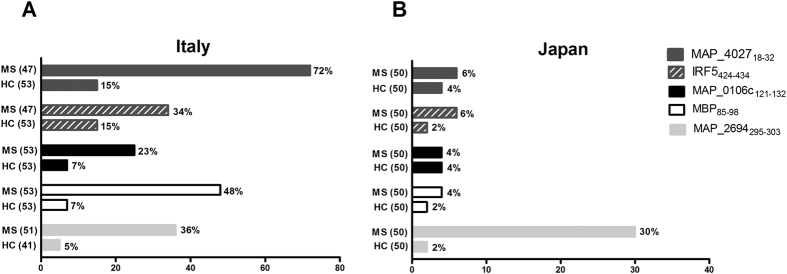
Distribution of antibody titers against MAP and human homologues peptides. Differences of the humoral response in MS patients and healthy controls from (**A**) Italy and (**B**) Japan.

All the results are summarized in Fig. 2. Analysis of Ab strongly depends on the antigen preparation and on detection method used, for this reason all the experimental protocols were performed following the same previously published methods^7^. The prevalence of Abs against MAP_2694_295–303_ peptide in Japanese MS patients (30%) was similar to the Italian study (35%).

The MAP_2694_295–303_ titers of the Japanese MS patients were 15 with a median [interquartile range] IgG titer of 2825 [2118–4885] AU/mL, while the MAP_2694_295–303_ titers of the Italian MS patients were 18 with a median [interquartile range] IgG titer of 1883 [1509–2873] AU/mL. The median Ab titer in Japanese HCs was 2452 [2155–2476] AU/mL, whereas in Italian HCs was 1284 [177–1560] AU/mL.

In contrast to the results obtained in Italy, no statistical significant reactivity against MAP_1026_121–132_ (23% vs. 4%), MAP_4027_18–32_ (72% vs. 6%), human homologues MBP_85-98_ (48% vs. 4%), and IRF5_424-434_ (34% vs. 6%) was detected in Japanese MS patients as compared to HCs.

The data highlighted the importance of MAP_2694_295-303_ as an immunogenic peptide that probably plays a role in determining immunogenicity in genetically susceptible MS patients. On the other hand, the discrepancies in Ab determination between our findings and published data, suggests that MS patients from different ethnic backgrounds have altered immune responses to MAP antigens.

Unfortunately, we could not perform a HLA typing and a comparison concerning the molecular detection of the mycobacterium by PCR, as DNA from MS Japanese patients was not available. However there is frequently a poor correlation between DNA detection and serological response to antigen, because their positivity can follow different pathways due to the immunological status (anergic or not) of the subjects^8^.

### Association between Ab-positivity and demographic/clinical characteristics

After comparing clinical features of MS patients there was evidence that oligoclonal bands (OCBs) were found with higher prevalence. Of the 15 MAP_2694_295-303_ positive MS patients we found that 10 (88%) patients were OCBs positive and 5 patients were OCBs negative (p = 0.2; OR = 2, 95%CI: 0.55–7.7). While at the present we cannot speculate as to what the exact role of the bacterium in triggering autoimmunity is, future CSF analyses will be interesting to confirm the direct role of bacteria in the CNS, since anti-MAP Abs must access the blood brain barrier in the CNS to infiltrate the brain and participate in tissue injury. As for these requirements, in paired serum/CSF samples, levels of anti-MAP Abs should correlate well between the two compartments, and should be measured around the time of an active episode of CNS inflammation.

Among the anti- MAP_2694_295-303_ positive MS patients (n = 15), the Ab levels were measured during acute symptomatic episodes of CNS inflammation in 3 patients (20%), while 12 patients (80%) had a stable disease activity.

There were no differences in the gender, mean age, age at onset, duration of disease, IgG index, anti-aquaporin 4 (AQP4) Abs or Expanded Disability Status Scale (EDSS) between MAP_2694_295-303_ seronegative and seropositive patients.

## Discussion

Autoreactive T cells have been shown to be a key mediator of the attack against the myelin sheath, and it has been hypothesized that an infectious pathogen might cause the activation of these cells, through a potential molecular mimicry mechanism^17^. Ongoing studies support the view that B cells are relevant in driving the pathologic immune-mediated response in MS^18^.

Recent studies conducted in Sardinia, have shown an association between MAP and MS^4^,^5^,^6^,^7^,^8^,^13^,^19^. With respect to the result of the above mentioned studies, in the current study only Abs against MAP_2694_295–303_ were more prevalent in MS patients compared to OND patients and HCs.

Antibodies against MAP_2694_295-303_ were also detected in a similar percentage of CIS patients. CIS is used to describe the first demyelinating episode of the CNS and can be indicative of several neurological diseases such as MS^20^. At present there is no diagnostic test that can establish whether a CIS will eventually develop clinically defined MS over time. For this reason it will be interesting to perform a follow-up study of these MAP-positive CIS patients, in order to see if MAP can be associated with a low or a high risk to developing clinically definite MS.

Interestingly, a certain percentage of MS patients had positive Abs for more than one peptide. The presence of Abs against epitopes-tagged no homologous regions in the sera of the same patient, can be explained because MS is influenced by intermolecular and intramolecular T and B cell epitope spreading^21^. In the phenomenon of B cell epitope spreading, additional Abs from the initial antigen that triggered the immune response develop against other endogenous epitopes of this antigen, or against completely new antigens^22^. This process can take place through different mechanisms, including interactions with T cells, endocytic processing of antigens and somatic hypermutation in the B cells^21^. Abs against MAP antigens which mimic self-antigen (i.e. gamma delta T cell receptor) may potentially be a contributor of B cell epitope spreading in same individual genetically susceptible. This mechanism might break self-tolerance and initiate the development of autoimmune disease.

A number of OND and HCs were also positive for different MAP peptides. The results are quite similar with the Ab-prevalence found in Italy [9.8% (5–14%)] and in agreement with the hypothesis that Japanese population is at risk to be exposed to MAP^11^.

There are multiple variants that can cause the differences between the two populations. The genetic background remains an important determinant of susceptibility of the disease, and Japanese and Caucasian MS subjects differ somewhat in their genomic structure. In Caucasians, the HLA-DRB1*1501 risk allele has the strongest association with MS susceptibility^23^. The general MS risk profile of the Sardinian population is similar to others outbred in European populations, there is a high frequency of several genetic variants that are rare in Europe mainly because of genetic drift^24^. HLA DPB1*0301 and in particular HLA DRB1*0405 alleles are associated with a greater risk of developing MS in a few countries such as Sardinia and Japan^10^,^25^. Sardinian MS patients showed a presence of *0301-*0201 and *0405-*0301 haplotypes in 75% and 18.75% of the MAP_2694_295–303_-positive subjects (Supplementary Table 1), respectively. Even if the HLA status of Japanese MS patients of this study is currently not available, we can speculate that the same genetic loci are associated with increased susceptibility to MS in MAP_2694_295-303_ positive subjects.

Another factor to take into account is the different assessment of human exposure to the mycobacterium, which is much lower in Japan than in Sardinia, where MAP is endemic and the rate of MAP infection is almost 60% among livestock^4^.

Lastly, it is also important to consider the different coverage rates of BCG vaccination between populations. BCG vaccine is compulsory in Japan, while in Italy the BCG vaccine is obligatory only for high-risk persons and specific professionals such as health care or military personnel. BCG immunization could provide protection against MAP, due to the presence of cross-reactive mycobacterial and self-epitopes^26^.

Overall, these findings support the complexity of MS disease, and highlighted that several non-mutually exclusive environmental risk factors can contribute to its etiology. Considering that MAP_2694_295–303_ displayed a preferential binding to the HLA predisposing proteins in the Sardinia population^27^, further exploration of the role of MAP in MS, in particular focusing on the T cell response elicited by its epitopes, would also generate evidence to further validate or refute MAP involvement in MS. In fact, the association of an infectious agent in the etiology/progression of a disease could be population dependent, and the relationship between MAP and MS could well be a population-specific phenomenon, highly dependent on different genetic and non-genetic factors.

## Methods

### Ethical considerations

The study protocol was approved by the National Ethical Committee of the Juntendo University School of Medicine (Approved No. 205), the Ethics Committee of Sangenjyaya Hospital (Approved No. 2014.3.7) and Tohto College of Health Sciences (Approved No. H2511). All the methods were carried out in “accordance” with the approved guidelines. All subjects provided written informed consent for participation.

## Subjects

The demographic and clinical data of all the subjects enrolled in this study, ethnically all Japanese, are shown in Table 1. For each patient, the data collected included gender, age, type of MS and duration of symptoms, family history for autoimmune diseases, neurological signs, hematological values, and presence of OCBs in the cerebrospinal fluid, IgG index, EDSS score, anti-AQP4 Abs and therapy.

A total of 50 MS patients (F:M ratio = 2.8:1) were assessed according to McDonald criteria 2010 for the diagnosis of MS^28^. The clinical characteristic of the MS patients were as follow: 44 (88%) relapsing remitting, 4 (8%) secondary progressive, and 2 (4%) primary progressive. None of the MS patients received any kind of immune/inflammatory-related treatment at the time of sampling collection.

In addition, 12 patients with CIS (F:M ratio = 4:1) without MS clinical features were recruited. The CIS group was defined by cases with all single inflammatory demyelinating episodes except neuromyelitis optica spectrum disorder.

Thirty patients (F:M ratio = 1.7:1) affected with OND including cerebrovascular diseases (4), peripheral nervous system disorders (5), migraine (7), spinal stenosis (2), Fisher and Guillain-Barre syndrome (2), myasthenia gravis (2), cervical myelopathy (1), stroke (1), HTLV-I associated myelopathy (1), chronic inflammatory demyelinating polyneuropathy (1), epilepsy (2) and dementia (2) were included in the disease control group.

Fifty sera from age and gender-matched HCs (F:M ratio = 2.8:1) with no history of autoimmune diseases were obtained for the healthy control group.

### Mycobacterial and human homologues peptides

Five peptides previously described as MS-related were used in this study (Table 2). MAP_0106c_121-132_ shares conformational homology with human MBP_85-98_ 6, while MAP_4027_18-32_ and human IRF5_424-434_ are linear homologues epitopes^7^. MAP_2694_295-303_, is an immunodominant epitope within MAP_2694 protein, a specific transmembrane protein of the mycobacterium that shares sequence homology with human gamma delta T cell receptor^8^,^13^. Synthetized peptides were purchased by GenScript (Piscataway, NJ, USA) at >90% purity. All peptides were dissolved in an appropriate solvent at a peptide concentration of [10 mM] and stored in single-use aliquots at −80 °C until use. All peptides were used at final concentration of 10 μg/mL for ELISA assays.

### Anti-AQP4 antibody cell-based assay

AQP4 Ab-detection were performed on all the patients’ sera as previously published^29^. Briefly, AQP4 live transfected human embryonic kidney cells (HEK293) expressing the untagged AQP4-M23 isoform were incubated in 4x diluted serum for 1 h, washed and incubated in fluorescein-conjugated goat anti-human IgG (MP Biomedicals, Aurora, OH, USA) for 30 min. Cells were fixed in 95% ethanol and mounted in the prolong antifade mounted media Permafluor (Beckman Coulter, Fullerton, Ca, USA). Image were captured by confocal microscopy Fluoview (Olympus Tokyo). Ab-seropositivity was based on comparison with mock-transfected cells that did not express AQP4.

### Enzyme-linked immunosorbent assay (ELISA)

Analyses of the levels of total IgG and IgG subclass in serum samples were carried out in triplicate wells by indirect ELISA. Nunc-immuno -MicroWell- 96 well solid plates (Thermo Fisher Scientific, Waltham, MA, USA) were coated overnight at +4 °C with optimal concentration of each antigen in ELISA coating buffer (Bio-rad, Tokyo Japan). The day after, plates were blocked with 200 μl of Blocking One (Nakalai tesque, Kyoto, Japan) and were incubated for 1 h at room temperature. After being rinsed with PBS-T ([10 mM] PBS, pH 7.0, containing 0.5% Tween 80) four times, serum samples were added at 1:100 dilutions in Blocking One for 2 h at room temperature. After washing as stated above horseradish peroxidase (HRP)-labeled-goat anti-human IgG polyclonal Ab (1/2000 dilution), anti-human IgG_1_-HRP (1:2000), or anti-human IgG_2_-HRP (1:2000), anti-human IgG3-HRP (1:4000), or anti-human IgG_4_-HRP (1:2000) (all purchased from Southern Biotech Associates, Inc., Birmingham, AL, USA), were added to each well and incubated for 1 h at room temperature. After the wash steps were repeated, 100 ml of ABTS Peroxidase System (SeraCare Life Sciences, KPL, Gaithersburg, MD, USA) was added to the wells and the reaction was allowed to proceed for 5–10 min in the dark at room temperature. Plates were read at optical density 650 nm for absorbance on a Benchmark Plus Microplate Reader (Bio-Rad, Tokyo, Japan). The results were normalized to a positive control serum included in all experiments, the reactivity of which was set at 10.000 arbitrary units (AU)/ml. Negative controls were obtained by incubation of immobilized peptides with secondary Ab alone, and their mean values were subtracted from all samples.

## Statistics

All analyses were performed using Graphpad version 6.0 software (San Diego, CA, USA). Comparisons between MS patients and HCs were performed using Mann Whitney U test. The level of statistical significance was set at p < 0.05. Area under the receiver operating characteristic curve (AUC) was calculated for all antigens, to determine the performance of each single ELISA in discriminating MS patients from HCs in each single ELISA. The cut-off for positivity in each assay was set at 95% specificity and the corresponding sensitivity calculated accordingly. Logistic regression analysis was performed to evaluate MAP positivity with respect to the following independent variables: gender, age, age at onset, OCBs, IgG index, EDSS and anti-AQP4 Abs.

Correlation analyses between Ab-titers of all peptides were calculated in MS group by the Spearman correlation test.

## Additional Information

**How to cite this article**: Cossu, D. *et al*. Humoral response against host-mimetic homologous epitopes of *Mycobacterium avium* subsp. *paratuberculosis* in Japanese multiple sclerosis patients. *Sci. Rep.* **6**, 29227; doi: 10.1038/srep29227 (2016).

## Supplementary Material

Supplementary Information

## Figures and Tables

**Table 1:** Demographic, clinical and serological characteristics of the cohort study.

	MS (50)	CIS (12)	OND (30)	HCs (50)
Gender: F/M	37/13	8/2	19/11	37/13
Age, mean ± SD, years	41.0 ± 11.2	39.6 ± 9.1	53.2 ± 19.6	41.2 ± 11.6
Age at onset, mean ± SD, years	32.7 ± 9.9	33.7 ± 7.8	45.8 ± 19.9	
Duration of disease, mean ± SD, years	8.36 ± 6.8	5.8 ± 5.7	5.3 ± 6.2	
Oligoclonal bands positive/tested (%)	31/47 (66)	0/7 (0)	3/24 (12.5)	
IgG index ≥ 0.7 (%)	30/44 (68)	1/7 (14)	6/24 (25)	
AQP4 Ab positive (%)	0 (0)	0 (0)	0 (0)	
EDSS score at onset, median (range)	2 (0−7)	1 (0−2)	3 (0−3.5)	
MAP_0106c_121-132_ IgG positive (%)	2 (4)	1 (8)	1 (3)	2 (4)
MBP_85-98_ IgG positive (%)	2 (4)	1 (8)	0 (0)	1 (2)
MAP_4027_18-32_ IgG positive (%)	3 (6)	1 (8)	4 (13)	2 (4)
IRF5_424-434_ IgG positive (%)	3 (6)	1 (8)	2 (6)	1 (2)
MAP_2694_295-303_ IgG positive (%)	15 (30)*	3 (25)*	1 (3)	1 (2)

MS, multiple sclerosis; CIS, clinically isolated syndrome; OND, other neurological diseases; HCS, healthy controls; EDSS, Expanded Disability Status Scale; *p < 0.05.

**Table 2:** Peptides selected for ELISA assays.

Protein	Peptide Sequence	Position	Organism	Reference
MAP_0106c	PGRRPFTRKELQ	121-132	MAP	6
Myelin basic protein	ENPVVNFFKNIVTP	85-98	Human	6
MAP_4027	AVVPVLAYAAARLLL	18-32	MAP	7
Interferon regulatory factor 5	VVPVAARLLLE	424-434	Human	7
MAP_2694	ADVTIADPT	295-303	MAP	13

## References

[b1] Belbasis L., Bellou V., Evangelou E., Ioannidis J. P. & Tzoulaki I. Environmental risk factors and multiple sclerosis: an umbrella review of systematic reviews and meta-analyses. Lancet Neurol 14, 263–273, doi: 10.1016/S1474-4422(14)70267-4 (2015). 25662901

[b2] Nguyen M. D., Julien J. P. & Rivest S. Innate immunity: the missing link in neuroprotection and neurodegeneration? Nat Rev Neurosci 3, 216–227, doi: 10.1038/nrn752 (2002). 11994753

[b3] Simpson S. Jr., Taylor B. V. & van der Mei I. The role of epidemiology in MS research: Past successes, current challenges and future potential. Mult Scler 21, 969–977, doi: 10.1177/1352458515574896 (2015). 25767125

[b4] Cossu D., Masala S. & Sechi L. A. A Sardinian map for multiple sclerosis. Future Microbiol 8, 223–232, doi: 10.2217/fmb.12.135 (2013). 23374127

[b5] Cossu D. . Are Mycobacterium avium subsp. paratuberculosis and Epstein-Barr virus triggers of multiple sclerosis in Sardinia? Mult Scler 18, 1181–1184, doi: 10.1177/1352458511433430 (2012). 22261119

[b6] Mameli G. . Epstein-Barr virus and Mycobacterium avium subsp. paratuberculosis peptides are cross recognized by anti-myelin basic protein antibodies in multiple sclerosis patients. J Neuroimmunol 270, 51–55, doi: 10.1016/j.jneuroim.2014.02.013 (2014). 24642384

[b7] Cossu D. . Human interferon regulatory factor 5 homologous epitopes of Epstein-Barr virus and Mycobacterium avium subsp. paratuberculosis induce a specific humoral and cellular immune response in multiple sclerosis patients. Mult Scler 21, 984–995, doi: 10.1177/1352458514557304 (2015). 25392335

[b8] Cossu D. . Association of Mycobacterium avium subsp. paratuberculosis with multiple sclerosis in Sardinian patients. PLoS One 6, e18482, doi: 10.1371/journal.pone.0018482 (2011). 21533236 PMC3076380

[b9] Dell’Avvento S., Sotgiu M. A., Manca S., Sotgiu G. & Sotgiu S. Epidemiology of multiple sclerosis in the pediatric population of Sardinia, Italy. Eur J Pediatr, doi: 10.1007/s00431-015-2588-3 (2015). 26156052

[b10] Yoshimura S. . Genetic and infectious profiles of Japanese multiple sclerosis patients. PLoS One 7, e48592, doi: 10.1371/journal.pone.0048592 (2012). 23152786 PMC3494689

[b11] Otsubo S. . Seroprevalence of IgG1 and IgG4 class antibodies against Mycobacterium avium subsp. paratuberculosis in Japanese population. Foodborne Pathog Dis 12, 851–856, doi: 10.1089/fpd.2015.1956 (2015). 26267654

[b12] Niino M. . Latitude and HLA-DRB1 alleles independently affect the emergence of cerebrospinal fluid IgG abnormality in multiple sclerosis. Mult Scler 21, 1112–1120, doi: 10.1177/1352458514560924 (2015). 25583844

[b13] Cossu D. . Antigenic epitopes of MAP2694 homologous to T-cell receptor gamma-chain are highly recognized in multiple sclerosis Sardinian patients. Mol Immunol 57, 138–140, doi: 10.1016/j.molimm.2013.09.001 (2014). 24091296

[b14] Hussain R., Shiratsuchi H., Phillips M., Ellner J. & Wallis R. S. Opsonizing antibodies (IgG1) up-regulate monocyte proinflammatory cytokines tumour necrosis factor-alpha (TNF-alpha) and IL-6 but not anti-inflammatory cytokine IL-10 in mycobacterial antigen-stimulated monocytes-implications for pathogenesis. Clin Exp Immunol 123, 210–218 (2001). 11207650 10.1046/j.1365-2249.2001.01439.x PMC1905980

[b15] Basile E., Gibbs E., Aziz T. & Oger J. During 3 years treatment of primary progressive multiple sclerosis with glatiramer acetate, specific antibodies switch from IgG1 to IgG4. J Neuroimmunol 177, 161–166, doi: 10.1016/j.jneuroim.2006.04.024 (2006). 16765453

[b16] Market E. & Papavasiliou F. N. V(D)J recombination and the evolution of the adaptive immune system. PLoS Biol 1, E16, doi: 10.1371/journal.pbio.0000016 (2003). 14551913 PMC212695

[b17] Chastain E. M. & Miller S. D. Molecular mimicry as an inducing trigger for CNS autoimmune demyelinating disease. Immunol Rev 245, 227–238, doi: 10.1111/j.1600-065X.2011.01076.x (2012). 22168423 PMC3586283

[b18] Hohlfeld R., Dornmair K., Meinl E. & Wekerle H. The search for the target antigens of multiple sclerosis, part 2: CD8+ T cells, B cells, and antibodies in the focus of reverse-translational research. Lancet Neurol 15, 317–331, doi: 10.1016/S1474-4422(15)00313-0 (2016). 26724102

[b19] Frau J. . Mycobacterium avium subsp. paratuberculosis and multiple sclerosis in Sardinian patients: epidemiology and clinical features. Mult Scler 19, 1437–1442, doi: 10.1177/1352458513477926 (2013). 23439580

[b20] Makshakov G. . Diagnostic and Prognostic Value of the Cerebrospinal Fluid Concentration of Immunoglobulin Free Light Chains in Clinically Isolated Syndrome with Conversion to Multiple Sclerosis. PLoS One 10, e0143375, doi: 10.1371/journal.pone.0143375 (2015). 26606531 PMC4659555

[b21] Cornaby C. . B cell epitope spreading: mechanisms and contribution to autoimmune diseases. Immunol Lett 163, 56–68, doi: 10.1016/j.imlet.2014.11.001 (2015). 25445494

[b22] Vanderlugt C. L. & Miller S. D. Epitope spreading in immune-mediated diseases: implications for immunotherapy. Nat Rev Immunol 2, 85–95, doi: 10.1038/nri724 (2002). 11910899

[b23] Barizzone N. . The burden of multiple sclerosis variants in continental Italians and Sardinians. Mult Scler 21, 1385–1395, doi: 10.1177/1352458515596599 (2015). 26438306

[b24] Sidore C. . Genome sequencing elucidates Sardinian genetic architecture and augments association analyses for lipid and blood inflammatory markers. Nat Genet 47, 1272–1281, doi: 10.1038/ng.3368 (2015). 26366554 PMC4627508

[b25] Sotgiu S. . Multiple sclerosis and anti-Plasmodium falciparum innate immune response. J euroimmunol 185, 201–207, doi: 10.1016/j.jneuroim.2007.01.020 (2007). 17336397

[b26] Rani P. S. . Mycobacterium avium subsp. paratuberculosis is not discerned in diabetes mellitus patients in Hyderabad, India. Int J Med Microbiol 304, 620–625, doi: 10.1016/j.ijmm.2014.04.010 (2014). 24863528

[b27] Kumar A. . Dynamical insights into the differential characteristics of Mycobacterium avium subsp. paratuberculosis peptide binding to HLA-DRB1 proteins associated with multiple sclerosis. New Journal of Chemistry 39, 1355–1366, doi: 10.1039/C4NJ01903B (2015).

[b28] Polman C. H. . Diagnostic criteria for multiple sclerosis: 2010 revisions to the McDonald criteria. Ann Neurol 69, 292–302, doi: 10.1002/ana.22366 (2011). 21387374 PMC3084507

[b29] Shimizu Y. . Pregnancy-related relapse risk factors in women with anti-AQP4 antibody positivity and neuromyelitis optica spectrum disorder. Mult Scler, doi: 10.1177/1352458515583376 (2015). 25921053

