# Supplementary material for: Humoral response against host-mimetic homologous epitopes of Mycobacterium avium subsp. paratuberculosis in Japanese multiple sclerosis patients
Source: Sci Rep. 2016 Jun 30;6:29227. doi: 10.1038/srep29227 (PMC4928110; doi:10.1038/srep29227)
Supplement: Supplementary Information [file srep29227-s1.doc]

**Humoral response against host-mimetic homologous epitopes of *Mycobacterium avium* subsp. *paratuberculosis* in Japanese multiple sclerosis patients**

Davide Cossu^1^, Kazumasa Yokoyama^1^*, Leonardo Antonio Sechi^2^, Shigeru Otsubo^3^, Yuji Tomizawa^1^, Eiichi Momotani^4^ and Nobutaka Hattori^1^

^1^ Juntendo University School of Medicine, Department of Neurology, Tokyo, 113-8421, Japan

^2^ Sassari University, Department of Biomedical Sciences, Sassari, 07100, Italy

^3^ Sangenjaya hospital, Department of Blood Purification,Tokyo, 154-0024, Japan

^4^ Tohto College of Health Sciences, Department of Human-care, Saitama, 366-0052, Japan

^*^ Corresponding author email: kazumasa@juntendo.ac.jp

**Supplementary Table 1**: Genetic and clinical features of Sardinian MS patients

|  |  | **Haplotype DRB1-DQB1** | | |
| --- | --- | --- | --- | --- |
| **MS patients (n=33)** |  | *0301-*0201  (n=22) | *0405/*0301  (n=6) | Others*  (n=5) |
| Gender: F/M |  | 19/3 | 5/1 | 4/1 |
| Age, mean ± SD, years |  | 43.1 ± 7.1 | 38 ± 11.8 | 42.0 ± 10.2 |
| Type of MS: RR/SP/PP |  | 20/1/1 | 6/0/0 | 4/1/0 |
| EDSS score at onset, median |  | 3 (0-8) | 1 (0-2) | 2 (0-6) |
| MAP_2694_295-303_ IgG positive |  | 75% | 18.75% | 6.25% |

*others haplotypes: *0401-*0302, *0101-*0501, *1601-*0502, *0701-*0201, *0501-*0102

MS, multiple sclerosis; RR, relapsing remitting; SP, secondary progressive; PP, primary progressive; EDSS, Expanded Disability Status Scale.
